# Divergent Mechanisms of Cranial Suture Ossification in Normal Development and Pathologic Fusion

**DOI:** 10.1111/jcmm.71125

**Published:** 2026-04-07

**Authors:** Anvith Reddy, Sarah Qaddo, Penny Li, Barite Gautama, Erin Abbott, Yomna Dean, Anna Means, Michael Golinko, Christopher Bonfield, Wesley Thayer, Galen Perdikis, Matthew Pontell

**Affiliations:** ^1^ Vanderbilt University Nashville Tennessee USA; ^2^ Department of Plastic Surgery Vanderbilt University Medical Center Nashville Tennessee USA; ^3^ Division of Pediatric Plastic Surgery Monroe Carell Jr. Children's Hospital at Vanderbilt Nashville Tennessee USA; ^4^ Department of Neurosurgery Vanderbilt University Medical Center Nashville Tennessee USA

**Keywords:** bone formation, cranial development, cranial suture biology, craniosynostosis, ossification mechanisms, skull growth, stem and progenitor cells

## Abstract

Cranial sutures are dynamic growth sites that balance bone growth with mesenchymal patency to accommodate cranial expansion during development. While intramembranous ossification has traditionally been considered the default mechanism of suture fusion, accumulating evidence demonstrates that endochondral pathways might also play a significant role under both physiological and pathological conditions. In this review, we contrast normal developmental ossification processes with premature fusion in craniosynostosis, integrating histological, molecular, and imaging data. We highlight the context‐dependent nature of cranial suture biology, influenced by embryonic origin, local signalling gradients, and genetic perturbations. Recognizing divergent ossification mechanisms reframes our understanding of both normal and premature suture fusion and has clinical implications for mechanism‐specific therapeutic strategies. Finally, we outline areas for future investigation, including high‐resolution profiling of human sutures across developmental stages, to establish a normative framework for cranial suture biology and inform mechanism‐driven regenerative approaches.

## 1 Introduction

Cranial sutures are fibrous joints between the flat bones of the skull. They allow cranial expansion during brain growth and remain patent through childhood [1]. These sutures are the sites of osteogenesis in the growing skull and are critical for balanced expansion of the cranial vault. Each suture consists of osteogenic fronts separated by a narrow layer of undifferentiated mesenchyme, which permits bone growth at the edges while maintaining flexibility at the joint. Mechanical forces from the expanding brain and molecular signals from the dura mater orchestrate a balance between new bone formation at the bone fronts and suture patency. The suture mesenchyme harbours stem and progenitor cells that self‐renew and supply osteoblasts, thus sustaining cranial growth while keeping the suture open [2]. Craniosynostosis, the premature fusion of one or more sutures, disrupts skull growth and can lead to cranial deformities and elevated intracranial pressure [3]. This review contrasts the ossification processes that occur during normal suture development with pathological fusion in craniosynostosis with a focus on key histological evidence.

### 1.1 Types of Ossification

Cranial bone ossification and cranial suture ossification are mechanistically and spatially distinct processes. Primary ossification centres for the frontal, parietal, temporal, and occipital bones appear in utero and expand radially; their osteogenic fronts continue to deposit bone postnatally [4, 5, 6]. In the parietal bones, two primary ossification centres per side typically arise at the parietal eminence between gestational weeks 7 and 8, serving as the nidus for bone formation that proceeds radially towards the surrounding sutures [6, 7]. Similarly, the frontal bones develop from paired ossification centres near the frontal eminences, which expand outward towards the coronal and metopic sutures. This bidirectional growth contributes to the symmetrical shaping of the forehead and brow ridge [6, 8]. In contrast, the occipital bone is derived from multiple ossification centres corresponding to the interparietal, supraoccipital, and basioccipital components. These centres undergo complex fusion events during foetal development and fusion [4, 6].

Cranial bone formation occurs via two distinct forms of ossification: intramembranous and endochondral. Intramembranous ossification involves the direct differentiation of mesenchymal progenitor cells into osteoblasts that deposit bone matrix without a cartilage intermediate. This process predominates in the calvarial vault and facial skeleton. In contrast, endochondral ossification involves a cartilage scaffold that is later replaced by bone through a tightly regulated process of chondrocyte maturation, hypertrophy, vascular invasion, and osteoblast‐mediated remodeling. Although traditionally considered exclusive to long bones, recent evidence shows that endochondral ossification can also occur in specific cranial sutures under both physiological and pathological conditions. Behr et al. [9] demonstrated normal endochondral closure of the murine posterior‐frontal suture, whereas Ueharu et al. [10] reported bone morphogenic protein (BMP)‐driven fusion in a pathological mouse model.

Histologically, intramembranous ossification is characterized by osteoblasts depositing woven bone matrix rich in Runx2, Osterix, and COL1A1, with no cartilage precursor present ([11], 155,164–172; [12], 232–238). In contrast, endochondral ossification begins with a cartilage template populated by chondrocytes expressing Sox9, COL2A1, COL10A1, and MMP9, then proceeds through hypertrophy and vascular invasion ([13], 232–238).

Ossification of the cranial suture itself occurs later in life and is characterized by the eventual bridging and mineralization of the suture [1, 14]. The foundational framework for understanding ossification in cranial sutures comes from classical histological studies of bone development. Pritchard et al. [15] originally delineated distinct zones within cranial sutures (capsular and cambial periosteum, osteogenic fronts, midline mesenchyme, and the underlying dura). Although influential, these early characterizations were rarely validated using modern techniques in well‐preserved infant human tissues. Furthermore, the ossification route in many sutures was assumed rather than directly demonstrated, and newer evidence now complicates these binary distinctions.

### 1.2 Timeline of Suture Fusion in Humans

**FIGURE 1:**
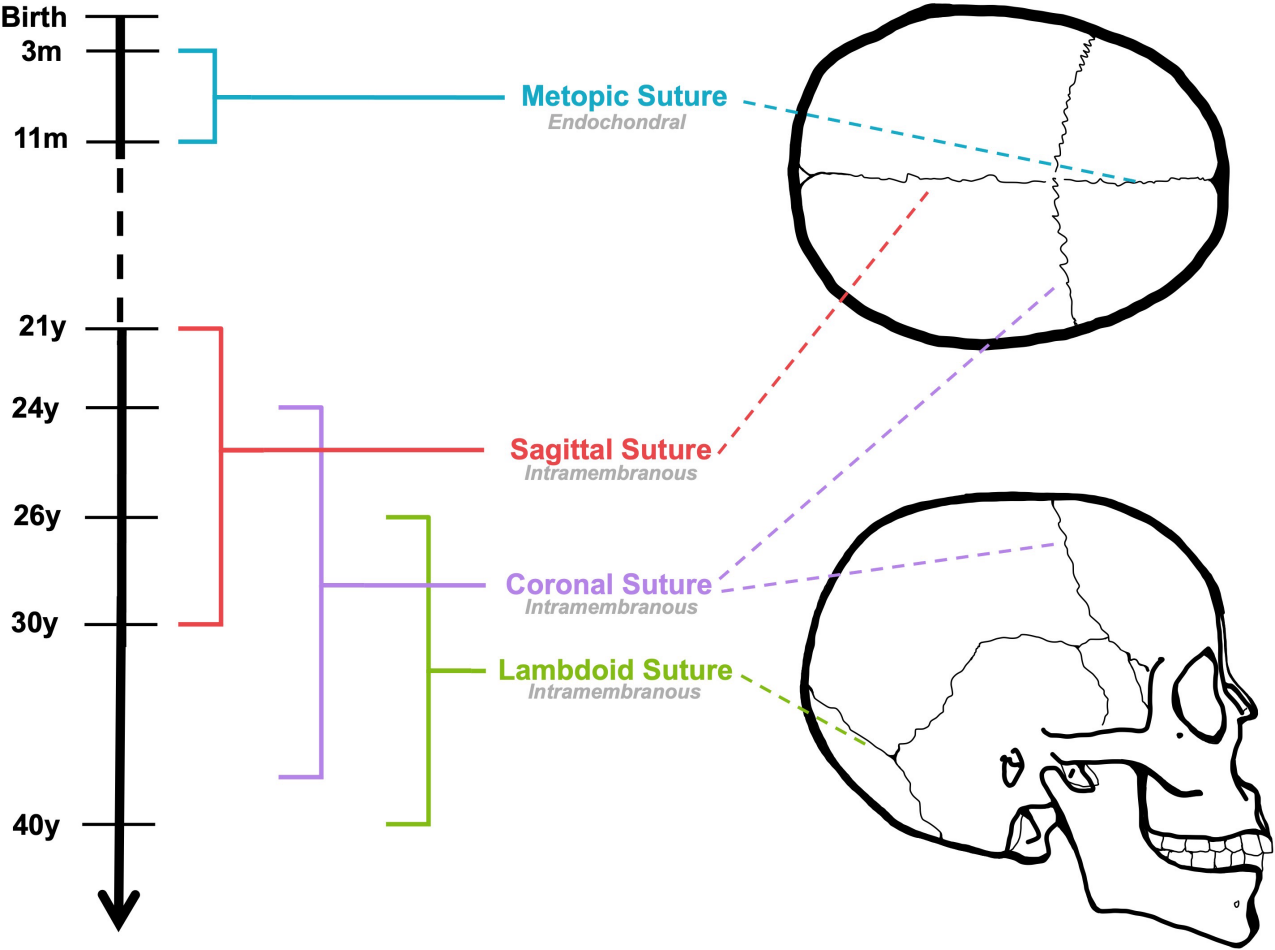
Schematic representation of the approximate timing of physiological cranial suture fusion. The metopic suture undergoes endochondral ossification and typically fuses during early childhood. The sagittal, coronal, and lambdoid sutures develop through intramembranous ossification, with fusion occurring between the third and fourth decades of life. Coloured dashed lines indicate the anatomical location of each suture on cranial views.

The timing and pattern of cranial suture fusion in humans follow a highly regulated, site‐specific, and prolonged developmental timeline (Figure 1). The metopic suture, between the frontal bones, is the only major calvarial suture that fuses during early infancy. Fusion of this suture typically begins around 2 to 3 months of age and is completed between 6 and 9 months. A large CT study showed that 33% of infants exhibit partial metopic closure by 3 months and 100% by 9 months [16]. Even so, complete fusion can be delayed, with some children achieving closure as late as 12–24 months, depending on genetic and environmental factors [17]. By contrast, the sagittal, coronal, and lambdoid sutures generally remain patent throughout childhood and adolescence. These sutures typically begin their fusion process in the third to fifth decade of life, often starting endocranially and progressing outward in an ectocranial direction [1].

Importantly, there is substantial inter‐individual variability in the timing of fusion, and in some individuals, certain sutures, like the sagittal and lambdoid, may never fully ossify. In a head‐CT series of 77 adults (18–101 years old), Soliman et al. found complete metopic fusion in 93.5% of patients, but complete fusion of the sagittal, squamosal, coronal, and lambdoid sutures in only 6.5%, 5.8%, 2.6%, and 0.7%, respectively. These data contradict the traditional view that all calvarial sutures close by mid‐adulthood and suggest that most remain at least partially patent throughout life [18]. This prolonged patency of sutures is essential for accommodating postnatal brain growth, skull reshaping, and biomechanical stress dissipation. However, it remains unclear why many sutures remain patent decades after cranial growth has ceased. Further investigation is needed to parse out whether this reflects a vestigial developmental program, continued mechanical demands later in life, or active maintenance by mesenchymal progenitors.

## 2 Normal Fusion Mechanisms

### 2.1 Evidence for Intramembranous Ossification

The developmental fate and fusion mechanisms of cranial sutures are closely influenced by their embryonic origin and anatomical context. Most mesoderm‐derived calvarial sutures, including the sagittal, coronal, and lambdoid, remain open during infancy and fuse through intramembranous ossification. Intramembranous sutures show osteoid bridging without cartilage, with high expression of Runx2 and COL1A1 and absence of Sox9 or COL2A1 [19]. Quantitative CT scans of 159 infants confirm that the sagittal, coronal, and lambdoid sutures stay radiolucent (non‐ossified) throughout the first year of life, consistent with a delayed, cartilage‐free fusion program [16]. Another infant CT series adds similar normative data, reporting full patency of the sagittal and lambdoid sutures at three months and fewer than 10% exhibiting any closure by one year [20]. Single‐cell and spatial transcriptomics of the developing mouse coronal suture document Runx2^+^/Sp7^+^ osteogenic fronts advancing across a Sox9‐negative mid‐suture mesenchyme [21]. High‐resolution μCT and histology of late‐gestation human sagittal sutures display the same direct extension of osteoid spicules from the bone cortices with no cartilage in between [22]. Taken together, these imaging, molecular, biochemical, and histological findings point to direct intramembranous bone formation as the dominant mechanism of normal calvarial suture fusion.

### 2.2 Evidence for Endochondral Ossification

In contrast, neural crest‐derived metopic suture, located at the cranial midline, displays end‐to‐end bone apposition and undergoes endochondral ossification in both human and mouse studies. Secondary cartilage formation between the frontal bones is characterized by the expression of Sox9, COL2A1, and COL10A1, followed by chondrocyte hypertrophy and vascular invasion. Histological sections of the metopic suture during early infancy reveal zones of cartilage characterized by chondrocyte lacunae and proteoglycan‐rich matrix staining (Safranin O+, Alcian Blue+). Behr et al. [9] demonstrated this process in the mouse posterior‐frontal suture, a model of the human metopic suture. Behr et al. [23] similarly showed a cartilaginous bridge rich in Sox9 and type II collagen in Twist1^+/−^ coronal sutures, reinforcing the role of endochondral mechanisms in craniosynostosis.

CT‐based human findings strengthen this conclusion. Vu et al. [16] found that 33% of infants showed partial metopic fusion by 3 months and complete fusion by 9 months, whereas Weinzweig et al. [24] documented a progressive endocranial‐to‐ectocranial closure pattern in 76 infants, with near‐complete fusion between 6 and 9 months. In addition, Bok et al. [25] identified DDR2^+^ chondroprogenitor‐like cells within human metopic tissue, providing direct cellular evidence for an endochondral route in normal skull development.

## 3 Craniosynostosis and Pathologic Suture Fusion

### 3.1 Evidence for Intramembranous Ossification

In syndromic forms of craniosynostosis linked to activating mutations in fibroblast growth factor receptors (FGFRs), such as those found in Apert and Crouzon syndrome, suture fusion occurs through accelerated intramembranous ossification, with no intervening cartilage template ([26]; X. [19]). These sutures demonstrate direct bone bridging between osteogenic fronts without the presence of a cartilage intermediate. FGFR signalling, particularly via FGF2 and FGF4, enhances osteoblast proliferation and maturation, promoting early mineralization. Greenwald et al. [27] showed that overexpression of FGF2 or FGF4 in developing murine calvarial sutures induces premature synostosis. Local application of recombinant FGF2 in otherwise normal rat sutures likewise provokes premature, cartilage‐free fusion [28]. Pharmacologic inhibition of ERK, a downstream effector of FGFR signalling, delays or prevents suture fusion in cultured explants and in vivo FGFR2 mutant models [29]. Together, these studies identify amplified FGFR–ERK signalling as a key driver of intramembranous synostosis in FGFR‐related craniosynostosis.

### 3.2 Evidence for Endochondral Ossification

Several studies have proposed that cartilage formation may play a role in pathological cranial suture fusion. Cohen [30] described the occasional presence of secondary cartilage in craniosynostotic sutures, suggesting that aberrant chondrogenic differentiation within the suture mesenchyme may contribute to premature fusion. Although these cartilaginous foci are not universally present, their occurrence supports the hypothesis that some cases of craniosynostosis may involve an endochondral ossification‐like mechanism. However, Cohen also noted that it remains uncertain whether these regions represent a causative intermediate or arise secondarily due to altered mechanical or developmental cues.

Experimental studies have identified cartilage matrix proteins, proteoglycans, and type II and III collagens within cranial sutures of animal models, particularly during active phases of growth or fusion. Alberius and Johnell [31] localized cartilage‐associated proteoglycans at sutural margins in rats, and Yen et al. [32] reported that high levels of type III collagen, which is often associated with transient cartilage‐like extracellular matrices, correlate with periods of rapid sutural growth. These findings suggest that suture mesenchyme may transiently express chondroid features, particularly under pathological or experimentally altered conditions.

Evidence for an endochondral program is more clearly demonstrated in genetic models of craniosynostosis. In Twist1 haploinsufficiency as seen in Saethre‐Chotzen syndrome, sutures display pathological suture fusion via endochondral ossification. Mechanistically, Twist1 normally inhibits chondrogenic differentiation by regulating FGFR and TGF‐βa signalling in the suture mesenchyme. When Twist1 is reduced, cartilage formation is activated. This is supported by Connerney et al. [33], who showed that ectopically inhibiting downstream FGF signalling in Twist1^+^/^−^ mice prevented synostosis. Behr et al. [23] further confirmed an endochondral program in Twist1^+/−^ coronal sutures: the closing suture contained a cartilaginous bridge rich in Sox9 and type II collagen, a finding backed by up‐regulated chondrogenic gene expression.

Another example is when BMP signalling is hyperactivated in neural crest cells. Constitutive activation of BMPR1A drives cartilage formation inside normally fibrous sutures; the cartilage is soon replaced by bone, producing premature fusion [10]. Boosting Smad‐dependent BMP signalling in the same lineage triggers a similar cartilage‐first sequence that ends in early synostosis [34]. Similarly, deleting the GNAS gene (alpha subunit of the stimulatory G protein) lowers cAMP signalling and steers suture mesenchyme towards a chondrocytic fate, so heterotopic cartilage forms in sutures and fontanelles before undergoing endochondral ossification and fusion [35].

### 3.3 Dual and Context‐Dependent Fusion

Cranial suture ossification is increasingly recognized as a context‐dependent process. Accumulating evidence indicates that the same genetic perturbation can lead to divergent ossification pathways depending on the suture, highlighting the complex interplay between gene expression, spatial signalling gradients, mesenchymal composition, and embryonic lineage (Behr et al., 2013; Yen et al., 2011; [10]).

For example, neural crest‐derived sutures such as the coronal and metopic may be more susceptible to chondrogenic programs, whereas mesoderm‐derived sutures, like the sagittal, default to intramembranous ossification [1, 33]. This lineage‐based plasticity is mirrored in regenerative and pathological settings, where frontal (neural crest‐derived) bones show different healing and fusion profiles compared to parietal (mesoderm‐derived) bones.

In mice, the posterior‐frontal suture normally undergoes physiological fusion via an endochondral process, requiring relatively low Wnt activity. However, in Axin2‐null mice, constitutive Wnt/β‐catenin results in persistent Sox9^+^/COL2A1^+^ cartilage and incomplete ossification [4, 5, 6]. In contrast, sagittal sutures in the same animals undergo premature fusion driven by excessive intramembranous bone formation, with a correlated increased expression of osteogenic markers like Runx2 and COL1A1. These findings underscore that Wnt signalling might act as a molecular toggle or switch [34, 36].

Furthermore, surgical specimens from FGFR2‐related syndromic synostosis reveal primarily intramembranous bone bridging. In Fgfr2^C342Y/+^ mice, activating mutations in cranial osteoprogenitors generate dense intramembranous bone across the coronal suture with minimal intervening cartilage [37]. In the Fgfr2^S252W/+^ Apert‐model, ectopic midline cartilage mineralizes and is replaced by bone, producing a hybrid fusion pattern that begins endochondrally and finishes with direct bone deposition [38]. These findings suggest either a mixed fusion mechanism or region‐specific transitions between ossification modes. Conversely, in Twist1 or BMP‐driven synostoses, which typically favour endochondral pathways, occasional zones of direct bone formation can be observed at the periphery of cartilage nodules [10].

### 3.4 Clinical and Therapeutic Implications

Understanding the mechanism of suture fusion has critical implications for targeted therapy and surgical planning. If craniosynostosis is driven by excessive intramembranous ossification, pharmacologic inhibitors of these pathways could offer potential adjunct therapies. For instance, Connerney et al. [33] demonstrated that administering an FGFR antagonist to Twist1^+^/^−^ mice after birth prevented coronal suture fusion. Similarly, in hyper‐Wnt models, Wnt modulators may help rebalance osteogenesis.

In contrast, pharmacological treatment might also be useful in premature endochondral ossification. BMP antagonists are increasingly recognized as regulators of chondrogenesis [39]. Therefore, the use of such antagonists might eventually play a role as adjunct therapy in mitigating premature endochondral ossification. In addition, Hedgehog pathway inhibition could also be a promising strategy. In GNAS‐deficient mice, Hedgehog blockade prevented heterotopic ossification, suggesting a potential non‐surgical intervention [40]. There already exists an FDA approved therapeutic targeting the Hedgehog pathway with numerous studies and ongoing clinical trials for advanced cancer treatment [41, 42, 43].

For these inhibitors to accurately function, it will be important to better classify ossification type. This highlights a future in which therapies could be customized to the patient's specific ossification mechanism.

## 4 Conclusion and Future Directions

Cranial suture fusion is a complex process governed by the interplay of anatomy, developmental timing, embryologic origin, and molecular signalling. While intramembranous ossification has long been considered the default pathway for calvarial bone and suture fusion, emerging evidence demonstrates that endochondral mechanisms may also contribute under specific physiological or pathological conditions.

Recent advances in single‐cell and spatial transcriptomics have begun to address these questions and unravel the heterogeneity within the cranial suture mesenchyme. These studies reveal distinct spatial domains of progenitor populations expressing osteogenic, chondrogenic, and fibrous markers, providing mechanistic insight into why ossification pathways may diverge across or even within sutures [25, 44]. However, much of this work remains confined to murine models. In human tissues, the precise sequence of cellular and molecular events within the suture is still poorly defined. Furthermore, the persistence of open sutures after the completion of cerebral expansion remains unexplained. Whether this prolonged patency reflects continued mechanical demand, active signalling from the dura, or intrinsic mesenchymal maintenance programs is unknown.

Moreover, the cellular and molecular distinctions between normally fused and pathologically fused sutures remain to be characterized in human specimens. Clarifying these mechanisms could inform future therapeutic strategies, including the potential for biologically targeted interventions to delay, prevent, or reverse premature suture closure.

To address these gaps, future studies must prioritize analyses of human cranial sutures across developmental time points and fusion states. Combining detailed histology with spatially resolved molecular profiling will be essential to establish a normative framework for cranial suture biology and skull development. Ultimately, defining the cellular and molecular mechanisms that govern suture fusion will be essential for shifting craniosynostosis care from purely surgical correction towards mechanism‐driven, regenerative strategies.

## Author Contributions

Conceptualization and methodology: Anvith Reddy, Anna Means, Wesley Thayer, Galen Perdikis, Matthew Pontell. investigation: Anvith Reddy, Anna Means, Galen Perdikis. supervision: Anna Means, Wesley Thayer, Galen Perdikis, Matthew Pontell. visualization: Erin Abbott. resources: Anna Means, Galen Perdikis. writing – original draft: Anvith Reddy, Sarah Qaddo, Penny Li, Barite Gautama, Erin Abbott, Yomna Dean, Anna Means, Michael Golinko, Christopher Bonfield, Wesley Thayer, Galen Perdikis, Matthew Pontell. writing – review and editing: Anvith Reddy, Sarah Qaddo, Penny Li, Barite Gautama, Erin Abbott, Yomna Dean, Anna Means, Michael Golinko, Christopher Bonfield, Wesley Thayer, Galen Perdikis, Matthew Pontell.

## Funding

The authors have nothing to report.

## Conflicts of Interest

The authors declare no conflicts of interest.

## Data Availability

Data sharing not applicable to this article as no datasets were generated or analysed during the current study.
